# Online hard example mining vs. fixed oversampling strategy for segmentation of new multiple sclerosis lesions from longitudinal FLAIR MRI

**DOI:** 10.3389/fnins.2022.1004050

**Published:** 2022-11-04

**Authors:** Marius Schmidt-Mengin, Théodore Soulier, Mariem Hamzaoui, Arya Yazdan-Panah, Benedetta Bodini, Nicholas Ayache, Bruno Stankoff, Olivier Colliot

**Affiliations:** ^1^Institut du Cerveau-Paris Brain Institute, Centre National de la Recherche Scientifique, Inria, Inserm, Assistance Publique-Hôpitaux de Paris, Hôpital de la Pitié Salpêtrière, Sorbonne Université, Paris, France; ^2^Institut du Cerveau-Paris Brain Institute, Centre National de la Recherche Scientifique, Inserm, Assistance Publique-Hôpitaux de Paris, Hôpital de la Pitié Salpêtrière, Sorbonne Université, Paris, France; ^3^Department of Neurology, Assistance Publique-Hôpitaux de Paris, Hôpital Saint-Antoine, Paris, France; ^4^Inria, Epione Project-Team, Sophia-Antipolis, France

**Keywords:** segmentation, deep learning, hard example mining, multiple sclerosis, MRI

## Abstract

Detecting new lesions is a key aspect of the radiological follow-up of patients with Multiple Sclerosis (MS), leading to eventual changes in their therapeutics. This paper presents our contribution to the MSSEG-2 MICCAI 2021 challenge. The challenge is focused on the segmentation of new MS lesions using two consecutive Fluid Attenuated Inversion Recovery (FLAIR) Magnetic Resonance Imaging (MRI). In other words, considering longitudinal data composed of two time points as input, the aim is to segment the lesional areas, which are present only in the follow-up scan and not in the baseline. The backbone of our segmentation method is a 3D UNet applied patch-wise to the images, and in which, to take into account both time points, we simply concatenate the baseline and follow-up images along the channel axis before passing them to the 3D UNet. Our key methodological contribution is the use of online hard example mining to address the challenge of class imbalance. Indeed, there are very few voxels belonging to new lesions which makes training deep-learning models difficult. Instead of using handcrafted priors like brain masks or multi-stage methods, we experiment with a novel modification to online hard example mining (OHEM), where we use an exponential moving average (i.e., its weights are updated with momentum) of the 3D UNet to mine hard examples. Using a moving average instead of the raw model should allow smoothing of its predictions and allow it to give more consistent feedback for OHEM.

## Introduction

Multiple Sclerosis (MS) is a chronic autoimmune demyelinating inflammatory disease of the central nervous system and represents the leading cause of non-traumatic motor disability of young people in Europe and North America (Howard et al., 2016). MS lesions, consisting of focal areas of demyelination, edema, and auto-immune inflammation, are visible on Magnetic Resonance Imaging (MRI), especially on Fluid Attenuated Inversion Recovery (FLAIR) as contiguous areas of hypersignal (Filippi et al., 2019). The decrease or absence of new FLAIR lesion formation over time is a key radiological endpoint in clinical trials assessing disease-modifying therapies in MS, and the absence of such radiological activity takes part in the “No Evidence of Disease Activity” score, used to monitor patient's disease control and to discuss potential therapeutic change at the individual level (Hegen et al., 2018). Novel lesion identification and segmentation is usually performed manually, or using semi-automated procedures, by radiologists or neurologists and is time-consuming and subject to intra- and inter-rater variability (Altay et al., 2013). The aim of the MICCAI MSSEG-2 challenge was to benchmark new automatic methods to segment new lesions based on two FLAIR MRIs from two longitudinal visits (baseline and follow-up) of the same patient. Already published methods for this task consists mostly of either non-deep learning methods (Cabezas et al., 2016) or deep learning methods using multiple MRI sequences (McKinley et al., 2020; Salem et al., 2020); there are very few deep learning methods for this precise task based uniquely on FLAIR sequences (Gessert et al., 2020). The present paper describes our contribution to the challenge. The backbone of our approach is a patch-wise 3D UNet (Çiçek et al., 2022). Our key methodological contribution is to introduce online hard example mining (Shrivastava et al., 2016) (OHEM) to tackle class imbalance. Indeed, one important characteristic of the dataset is that there are fewer voxels belonging to a new lesion (positive) than not belonging to a new lesion (negative), images comprise on average approximately 0.005% of positive voxels. Notably, we use a moving average of our 3D UNet to perform inference for hard example mining. Our goal is that, similar to He et al. (2020), doing so will provide more stable predictions as training progresses. The present paper extends that published in the proceedings of the MICCAI MSSEG-2 2021 workshop (Commowick et al., 2021) by providing a more extensive description of the methodology as well as more detailed experimental results including the testing of the algorithm on another cohort (Bodini et al., 2016) distinct from the MICCAI MSSEG-2 testing dataset.

## Methods

### Preprocessing

We resampled each FLAIR image to a voxel size of 0.5 mm as it is the highest resolution of the training dataset and applied a *z*-score normalization to each FLAIR individually. As the two consecutive FLAIR images (baseline and follow-up) of a patient have been aligned in the halfway space using a rigid transformation by the challenge providers, our method starts by concatenating them along the channel dimension, resulting in a tensor of shape 2^*^D^*^H^*^W, where D, H, and W are, respectively, the depth, height, and width of the resampled FLAIR image. This tensor is then subdivided into patches of shape 2^*^32^*^32^*^32, which are passed through a 3D UNet to obtain the segmentation.

### Model

Our backbone model is a standard 3D UNet, which can be described by the following equations:


$$\begin{array}{l} \text{B}\left(\text{n}\right)\;:\text{=2x }\{\text{3DConvolution}\left(\text{n}\right)\to \text{Group Normalization}\\ \to \text{ReLU}\}\\ \text{3D UNet }:\text{=B}\left(\text{16}\right)↓\;\to \text{B}\left(\text{32}\right)↓\to \text{B}\left(\text{64}\right)\to \\ ↑\text{B}\left(\text{32}\right)\to ↑\text{B}\left(\text{16}\right)\to \text{Conv}\left(\text{1}\right) \end{array}$$

where the numbers in the parentheses are the number of filters, ↓ indicates max pooling and ↑ indicates trilinear upsampling. The model is trained on patches of size 32. For inference, we split the image into a grid of patches of size 32, with a stride of 24. This means that the patches have an overlap of 8 pixels. In these overlapping regions, we averaged all predictions and binarized the final output with a threshold of 0.5.

### Dataset

We used the MICCAI MSSEG-2 datasets (Commowick et al., 2021) for training, validation, and the first testing set (see Appendix). We also used a second testing set consisting of a previously published MS cohort from our laboratory (Bodini et al., 2016). This cohort was constituted of 19 patients with active relapsing remitting MS (13 women, mean age 32.3 years sd 5.6) who underwent two MRIs with FLAIR spaced from minimum 31 days to maximum 120 days. Of those 19 patients, only 18 had available FLAIR MRIs for each visit. As only one of those 18 remaining patients had no new lesions at the second visit, we focused on the 17 patients that presented new lesions at the second visit for the second testing dataset. For these 17 patients, the new lesions at the second visit were manually contoured in native space and verified by a senior neurologist. After rigid co-registration to halfway space (FLIRT, http://fsl.fmrib.ox.ac.uk/) (Jenkinson and Smith, 2001), we gave the baseline and the follow-up FLAIR as input to our algorithm, and the manually contoured lesion mask as ground truth to evaluate our algorithm performances. Acquisitions for our testing cohort were run on a 3 Tesla Siemens machine, with a 32-channel head coil (Repetition Time: 8.88 ms; Echo Time: 129 ms; Inversion Time: 2.5 ms; Flip Angle: 120°; Pixel size: 0.9 × 0.9 × 3 mm).

### Training

**Figure 1:**
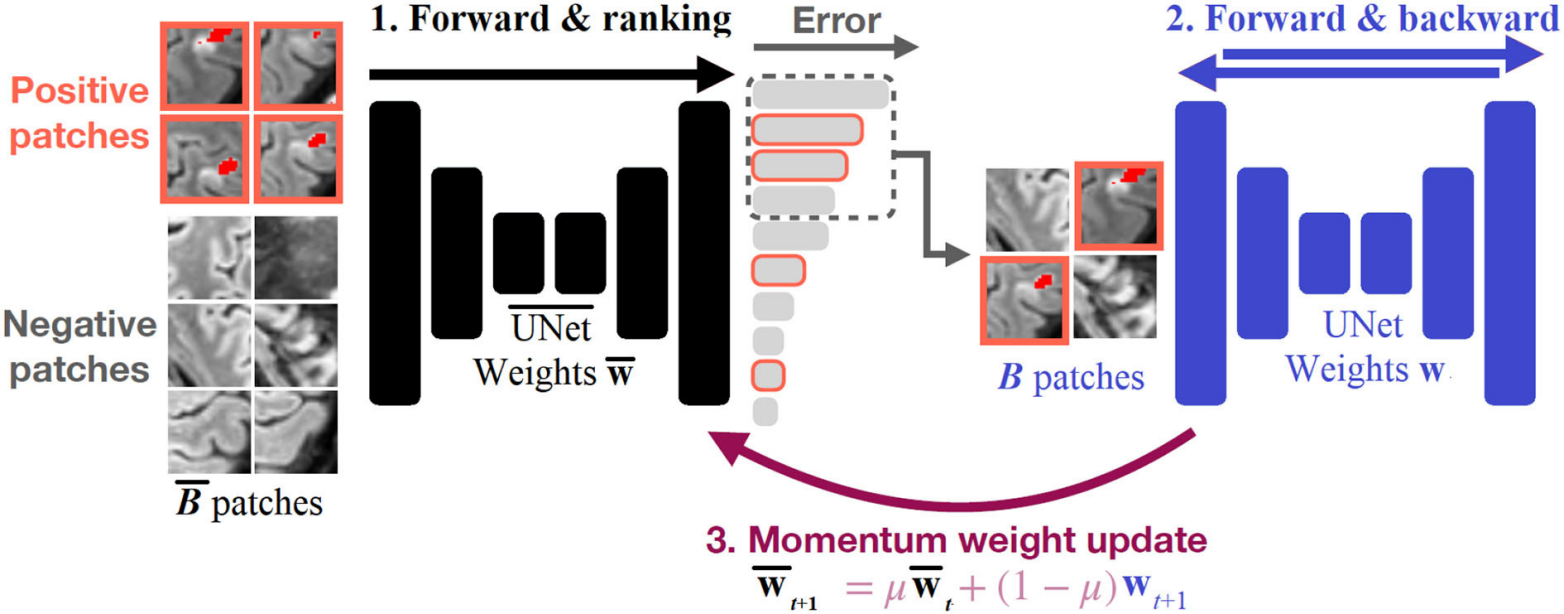
Illustration of our training strategy. $\bar{B}$ patches are fed to a first 3D $\bar{UNet}$ and the segmentation errors are computed for each patch. The patches are ranked according to their errors, and the top_B_ patches are selected to perform a training step with a second 3D *Unet*. The weights of the first 3D $\bar{UNet}$ are momentum-updated with the weights of the second 3D *Unet*.

As the images contain very few positive voxels, we do not sample the patches uniformly during training. One common strategy is to over-sample patches containing positive regions with a constant ratio. However, this ratio must be fine-tuned by hand. If it is too high, it can result in many false positives. Instead, our method uses a 3D UNet with momentum weight updates to perform hard example mining. A training iteration consists of three steps, illustrated in Figure 1 and described by Algorithm 1. In the first step, we select a batch of $\bar{B}$ = 128 patches, which contains 30% of positive patches and 70% of uniformly sampled patches (i.e., mostly negatives due to the class imbalance). We then pass this batch through a 1st 3D UNet, denoted by $\bar{UNet}$, to obtain a prediction for each element of the batch and compute the segmentation errors with respect to the ground truth. Second, we select the *B* = 32 patches with the highest error and perform a training step on them with a second 3D UNet, denoted *Unet*. Last, we perform a momentum update of the weights of the 1st 3D $\bar{UNet}$, with the second 3D *Unet*. The use of momentum ensures that the predictions given by the 1st 3D $\bar{UNet}$ do not fluctuate too much during training and provide reliable samples for online hard example mining.

**Algorithm 1:**
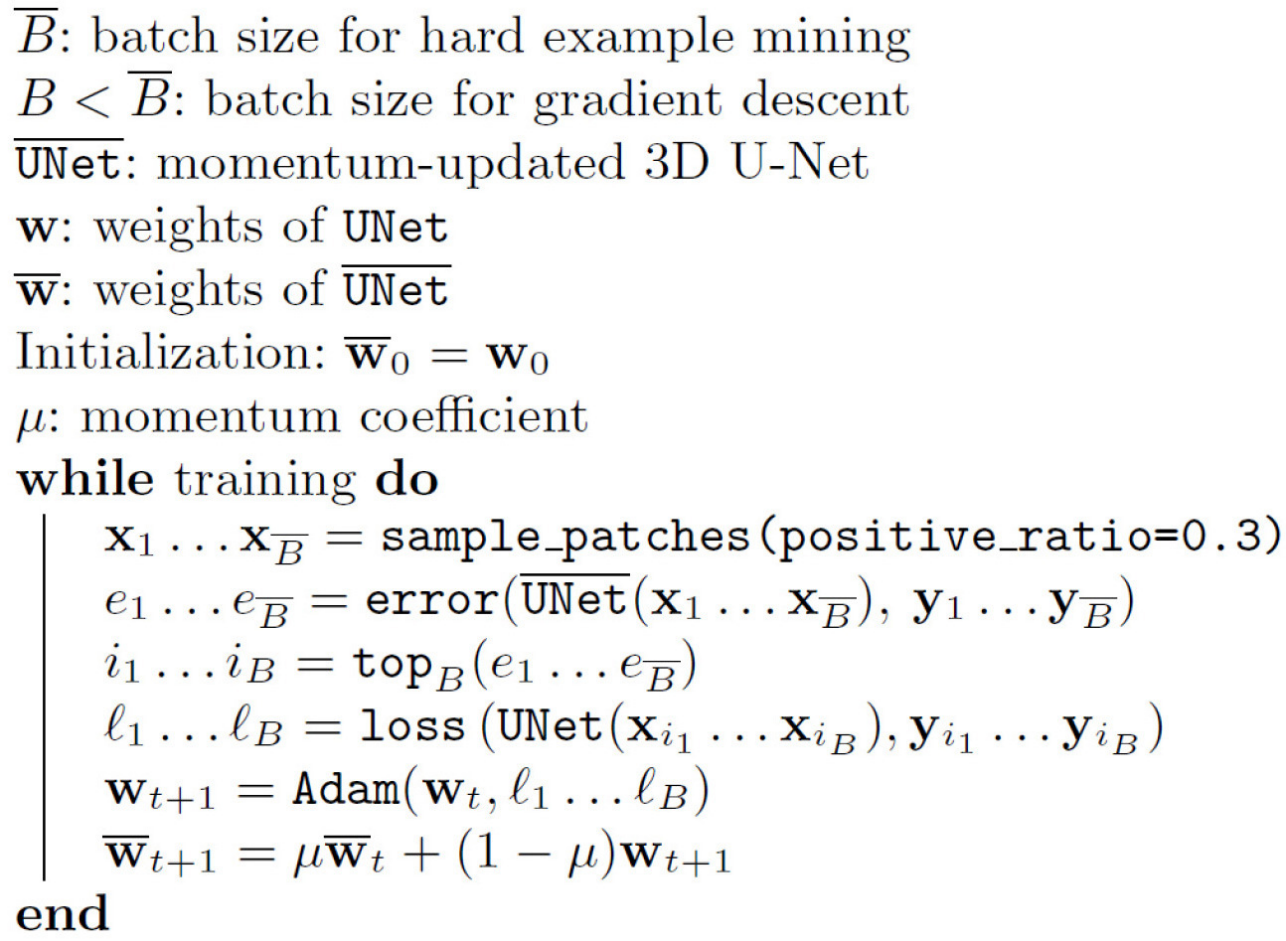
The algorithm used for the training with OHEM and momentum update.

### Training—OHEM vs. oversampling comparison

We optimized each network for 3 h on one NVIDIA Tesla P100 graphic card using Adam (Kingma and Ba, 2022). Note that for OHEM, the duration of one iteration is roughly 2 times longer. In the end, 3 h of training corresponds to about 30k iterations with OHEM and 64k without. The initial learning rate was set to 10^−3^ and decayed to 10^−4^ and 10^−5^ after, respectively, 50 and 80% of the training time. We split the dataset into 30 patients for training, and 10 for validation.

We compared the learning curves using the Dice score on the validation set for six training procedures: three with OHEM with a momentum of, respectively, 0, 0.9, and 0.99, and three without OHEM but with oversampling with a probability p of, respectively, 0 (uniform), 0.1, and 0.5. This oversampling probability meant that we sampled positive patches (i.e., with a new lesion at a second time point) with a probability p and other patches (that could be randomly positive or negative) with a probability 1-p for the training.

### Training—Final approach provided for the MSSEG-2 challenge

We used the model described before, using OHEM with a momentum of 0.9, and trained the model on the whole MICCAI MSSEG-2 training dataset for 30k iteration. As above, the initial learning rate is set to 10^−3^ and decayed to 10^−4^ and 10^−5^ after, respectively, 50 and 80% of the training time.

### Evaluation metrics for the testing dataset

The evaluation procedure was defined by the MICCAI MSSEG-2 committee (Commowick et al., 2021). We briefly recall this procedure in the following. The MICCAI MSSEG-2 testing dataset of 60 patients was divided into two subsets, according to the presence or absence of new lesions in patients: 28 patients without new lesions and 32 patients with new lesions. Those two datasets were evaluated differently.

All new lesions from the ground truth and our algorithm prediction were individualized by computing the connected components, and all lesions smaller than 3 mm^3^ were removed (Commowick et al., 2018). The detection was defined at the lesion level using the algorithm described by Commowick et al. (2018) with the parameters α = 10%, β = 65%, and γ = 70%, which were set by the MICCAI MSSEG-2 committee.

For the 28 patients without new lesions, the following metrics are reported: the lesion volume prediction per patient in mm^3^, and the new lesion detection rate per patient.

For the 32 patients with new lesions, the evaluation aimed at assessing both the quality of the detection and the segmentation. For evaluating the segmentation, the (voxel-level) Dice score per patient was reported. For evaluating the detection, the following metrics were used: the mean sensitivity *Sens* (=recall) at the lesion level per patient for detecting new lesions, and the mean positive predictive value *PPV* (=precision) at the lesion level per patient for detecting new lesions and the F_1_ score at the lesion level (which combines lesion-level *Sens* and *PPV*) per patient (Commowick et al., 2018).

The calculation of those metrics is described below. True positives with respect to the ground truth TP_gt_ were defined as the number of new lesions from the ground truth that were correctly detected by our algorithm. True positives with respect to our prediction TP_pred_ correspond to the number of new lesions predicted by our algorithm that were correctly detected by the ground truth.

- $Dice=\frac{2\;|PRED\cap GT|}{\left|PRED|+|GT\right|}$, where PRED is the network prediction and GT the ground truth segmentation, |*PRED*∩*GT*| is the number of overlapping voxels between the prediction and the ground truth, |*PRED*| is the number of voxels in the prediction and |*GT*| the number of voxels in the ground truth.
- $Sens=\;\frac{TP_{gt}}{n_{new\;lesions_{gt}}}$ where TP_gt_ and n_new lesions_gt_ are, respectively, the true positives with respect to the ground truth and the number of new lesions in the ground truth.
- $PPV=\;\frac{TP_{pred}}{n_{new\;lesions_{pred}}}$ where TP_pred_ and n_new lesions_pred_ are, respectively, the true positives with respect to our prediction and the number of new lesions in our prediction.
- $F_{1}=\frac{2^{*}Sens^{*}PPV}{Sens+PPV}$ where *Sens* and *PPV* are, respectively, the previously defined sensitivity and Positive Predictive Value.

All of those metrics were compared to zero for patients without new lesion, and to the ground truth segmentation of patients with new lesion, which is the consensual segmentation from four expert annotators (Commowick et al., 2021). All results are presented as mean, Standard Error to the Mean (SEM), and rank among other challenge pipelines when available.

For the second testing dataset, constituted by the 17 patients with new lesions in our cohort, we used exactly the same evaluation procedure that we described above for the patients with new lesions from the MICCAI MSSEG-2 testing dataset.

### Implementation details

Our algorithms were implemented on PyTorch (Paszke et al., 2017) and written using TorchIO library (Pérez-García et al., 2021). The implementation was based on that of Wolny et al. (2020). Training was performed on an NVIDIA Tesla P100 graphic card.

## Results

### Results on the validation set: Impact of the OHEM procedure

**Figure 2:**
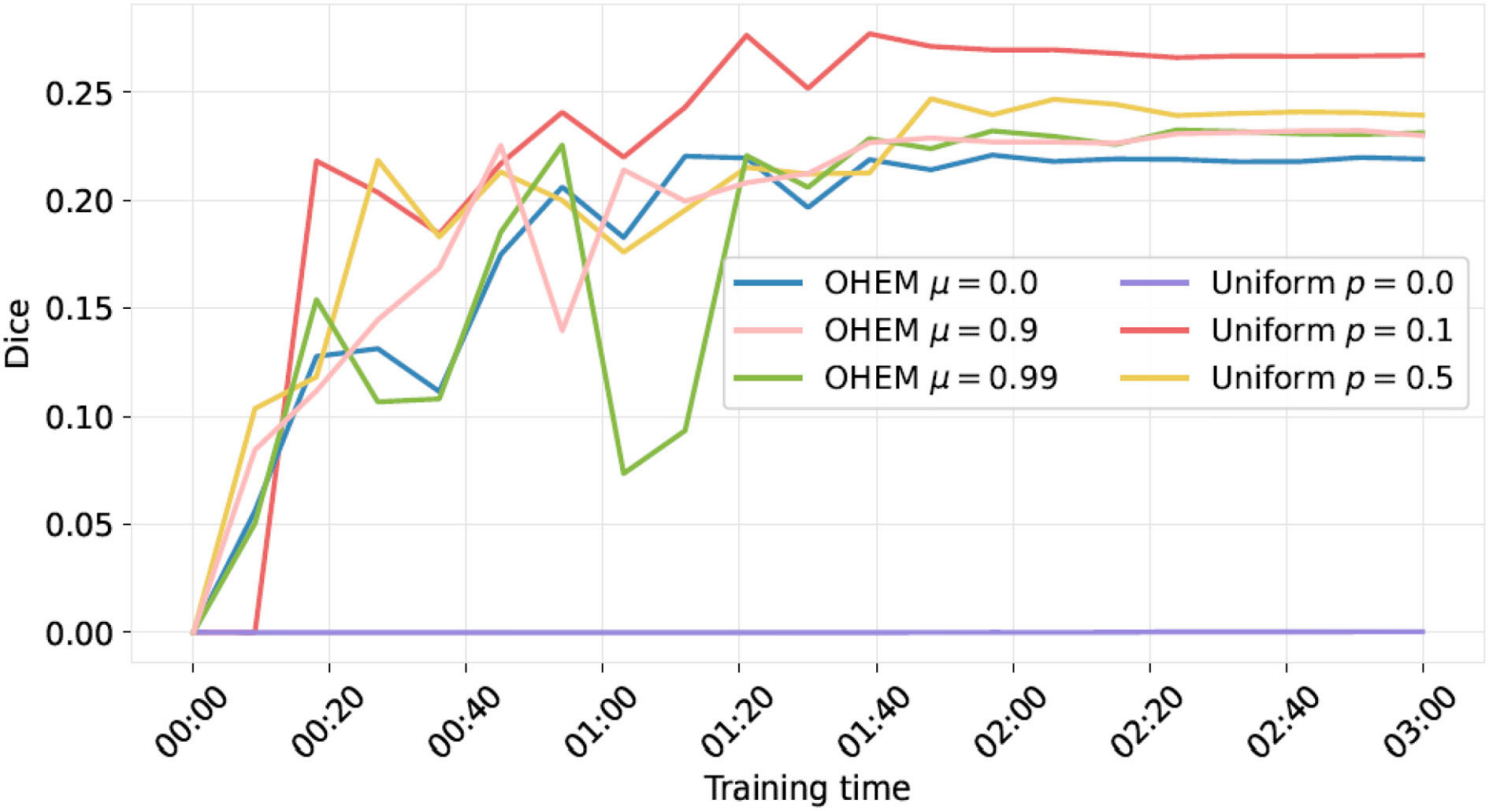
Evolution of the Dice score as a function of training time for the OHEM and the forced oversampling procedure (denoted as “Uniform”). For OHEM, μ is the momentum. For “Uniform”, patches were sampled with respective probabilities, p for those with new lesions, and 1-p for the rest (not necessarily without new lesions). One can observe that the “Uniform” procedure with *p* > 0 ended up performing best and that, when using OHEM, choosing μ > 0 seems to be beneficial.

The comparison of the learning curves for the proposed OHEM procedure and the forced oversampling procedure is shown in Figure 2. One can observe that, on this task, the OHEM procedure, even with increasing the momentum to 0.99, did not give better results in terms of training speed nor plateau of the Dice score. However, we observed that using OHEM gives a positive momentum that helped to reach a higher plateau of the Dice score on the validation set compared to a null one.

### Results on the testing set

Results on the first testing set from MICCAI MSSEG-2 are shown in Table 1. In the 32 patients with new lesions, our network achieved a mean lesion-level F_1_ score per patient of 0.446 (SEM 0.057), ranking 13th over 29 approaches for this metric. The mean Dice per patient was 0.400 (SEM 0.051), which ranked 18/29. Our mean sensitivity at the lesion level per patient was 0.616 (SEM 0.069) and our mean positive predictive value at the lesion level per patient was 0.383 (SEM 0.054). Concerning the 28 patients without new lesions, for whom any prediction is a pure false positive, on average, 0.75 (SEM 0.32) new lesions were predicted per patient (ranking our approach 15/29), with a mean lesion volume per patient among those 28 patients without new lesion of 31.2 mm^3^ (SEM 13.0), which corresponded to a rank of 20/29.

**Table 1:** Results on the testing set using MICCAI MSSEG-2 evaluation metrics, with the specific evaluation metrics from MICCAI MSSEG-2 testing dataset for the 32 patients with new lesions (a) as well as for the 28 patients without new lesions (b), and the 17 patients with new lesions from our second testing dataset (c).

**(a) MICCAI MSSEG-2 testing dataset: patients with new lesions (*n =* 32)**			
**Lesion-level F**_1_ **score per patient**, **mean (SEM); rank**	**Dice score per patient**, **mean (SEM); rank**	***Sens*** **at lesion level per patient**, **mean (SEM)**	***PPV*** **at lesion level per patient**, **mean (SEM)**
0.446 (0.057); 13^th^/29	0.400 (0.051); 18^th^/29	0.616 (0.069)	0.383 (0.054)
**(b) MICCAI MSSEG-2 testing dataset: patients without new lesion (*****n** =* **28)**			
**Number of new lesions predicted per patient**,		**Lesion volume predicted in mm**^3^ **per patient**,	
**mean (SEM); rank**		**mean (SEM); rank**	
0.750 (0.320); 15^th^/29		31.2 (13.0); 20^th^/29	
**(c) Second testing dataset: patients with new lesions (*****n** =* **17**)			
**Lesion-level F**_1_ **score per patient**, **mean (SEM)**	**Dice score per patient, mean (SEM)**	***Sens*** **at lesion level per patient**, **mean (SEM)**	***PPV*** **at lesion level per patient, mean (SEM)**
0.365 (0.038)	0.465 (0.046)	0.901 (0.043)	0.239 (0.030)

On our second testing set from our laboratory, on the 17 patients with new lesions, the mean Dice per patient was 0.465 (SEM 0.046). At the lesion level, our network achieved a mean sensitivity per patient of 0.901 (SEM 0.043) and a mean positive predictive value per patient of 0.239 (SEM 0.030), resulting in a mean lesion-level F_1_ score per patient of 0.365 (SEM 0.038).

**Figure 3:**
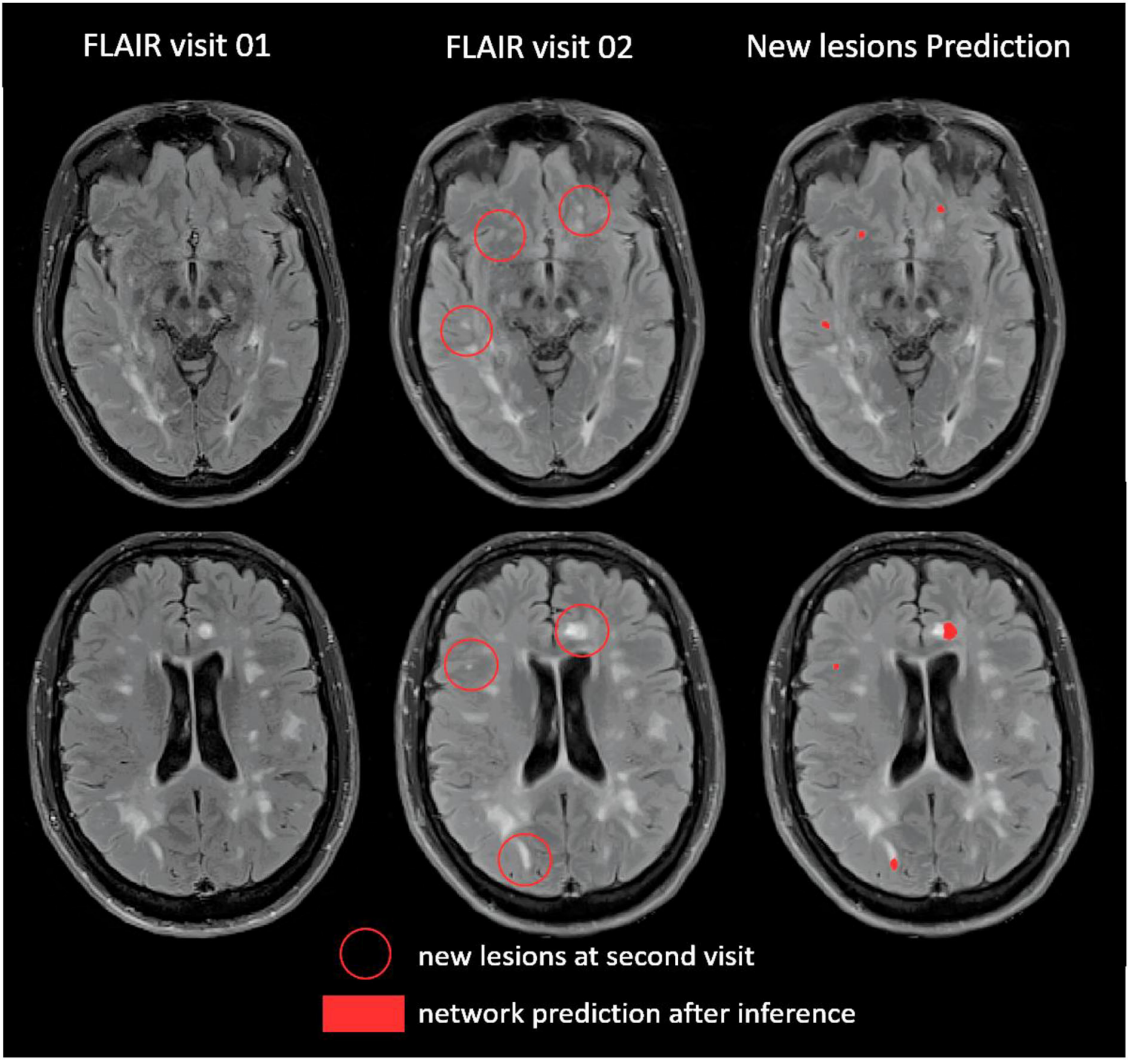
Example of prediction on one patient from our second testing dataset (Bodini et al., 2016).

Figure 3 shows an example of inference on a follow-up MRI from this second testing set from our laboratory.

## Discussion

The main contribution of this work was the introduction of online hard example mining (OHEM) to deal with class imbalance. The rest of the approach is constituted of a standard 3D UNet. We first showed that the use of a non-negative momentum helped the training procedure. However, overall, OHEM did not perform better than a predefined fixed oversampling and especially performed worse when an oversampling probability of *p* = 0.1 was used for fixed oversampling.

On the MICCAI MSSEG-2 testing set, our approach ranked in the mid-class of the challenge (Dice score of 0.400, corresponding rank 18/29; lesion-level F_1_ score of 0.446, rank 13/29). Interestingly, compared to other pipelines of the challenge, our worst performances were on the subset of patients without new lesions, where any prediction is a false positive. Together with the relatively high sensitivity but relatively low PPV, this could be explained by a bias in the OHEM training toward a high detection rate, resulting in a greater false positive rate. This trend was even stronger when we evaluated the algorithm performances on our second testing dataset, with a higher Dice score of 0.465, a higher sensitivity of 0.901 but a lower PPV of 0.239.

When compared to other pipelines of the challenge, the best pipeline in the subset of patients without new lesions, consisting of a 3D UNet with pre-activation block, also used an oversampling strategy for Regions of Interest with new lesions, but was also ranked in the mid-class of the challenge for the Dice score on the patients with new lesions (with a Dice score of 0.409). The most accurate pipeline in terms of Dice score (even better than several annotators), which did not use any oversampling strategy, was ranked in the mid-class of the challenge for the subset of patients without new lesions for the score of new lesions detection rate. This is consistent with the idea that dealing with the oversampling of positive examples is a key problem in the balance between false positive and false negative predictions in this new lesion segmentation task. We believe, given the medical utility of this task at the individual level for patient follow-up, that a compromise between sensitivity and PPV favoring sensitivity is clinically relevant if the algorithm is considered as an auxiliary to the neurologist or radiologist. Indeed, the interrater variability in manual new lesions detection is mainly explained by false negative rate (Altay et al., 2013), i.e., new lesions that were not detected by the rater. We believe that sensitive algorithms could help neurologists or radiologists to detect those overlooked new lesions. The clinicians could subsequently easily remove false positive predictions of the algorithm after visual checking. However, there is still a long way to go for clinical applications of algorithms for new lesion segmentation. This will require not only algorithm improvement but also prospective validation studies on larger and very diverse datasets.

There was only one pipeline in the challenge that did not use deep learning. Even if they outperformed four deep learning teams on average, their ranking was low on the MICCAI MSSEG-2 testing dataset, with a mean Dice of 0.309 for patients with new lesions, and a mean volume of new lesions detected of 177.9 mm^3^ for patients without new lesions. This does not mean that non-deep-learning methods are not potentially useful for this task but this would require additional comparisons which are outside of the scope of the present work. To our knowledge, most of the previously published deep learning algorithms (McKinley et al., 2020; Salem et al., 2020) or recent non deep learning based on deformation field (Cabezas et al., 2016) used to segment new lesions on MS MRIs are based on multiple MRI sequences and not only on a single sequence. It is the same when looking at previously published deep learning algorithms used to segment the lesion load transversally (Valverde et al., 2019; Zeng et al., 2020). So, even if clinically relevant (Hegen et al., 2018), the challenge task allows neural network to learn less information for prediction than in most of the state of art methods, and it can partly explain the difficulty of the task. The previous work from Gessert et al. (2020) based on attention gated two paths convolutional neural networks was to our knowledge the most relevant deep learning work published on the task of segmenting MS new lesion based only on two follow up FLAIR sequences. They did not require an oversampling procedure to deal with class imbalance and had very good lesion-wise true positive rate and lesion-wise false positive rate. However, we could not compare methods since their proposed evaluation metrics differed from the ones provided by MICCAI MSSEG-2 (Commowick et al., 2018).

This work has several limitations. First, concerning the OHEM training methodology (Shrivastava et al., 2016), it did not improve the training procedure on this task and did not outperform significantly other competing 3D UNets across the challenge. Despite being an interesting methodology to deal with class imbalance, we have to keep in mind that it has been developed for detection in 2D natural images (Shrivastava et al., 2016) using fast R-CNN (Wang et al., 2016). Even though it has shown promising results in Bian et al. (2022) work on heart MRI, unveiling its full potential for 3D medical image segmentation may require further adaptations and developments. Second, we chose to compare OHEM and fixed oversampling as a function of training time and not as a function of epochs. Training time could be influenced by many parameters like machine heat and GPU availability. However, we believe it was the fairest way to compare methods. Indeed, the unit cost of each epoch (or iteration) has no reason to be the same for the different techniques. Even worse, it can vary across epochs due to the nature of the OHEM method. Another limitation is that we used a single split into training and validation rather than a cross-validation strategy. Thus, we did not use all samples for testing and we did not assess their variability when varying the training set. We made this choice because we had to provide one single result for the challenge. We did not use data augmentation in our training strategy to be able to compare different oversampling strategies and momentum, but OHEM comportment should be explored with data augmentation in future work. Due to the short delay between baseline and follow-up MRIs in the MICCAI MSSEG-2 dataset (from 1 to 3 years) as well as in our second testing dataset (maximum 120 days), we could not explore the influence of severe atrophy in this task. An adjacent and clinically useful task for longitudinal follow-up of MS patients, that we could not assess here due to challenge constraints focusing on new lesions, is the detection of shrinking and enlarging lesions. Furthermore, it is likely that the use of multicontrast MRI could improve the results over the use of FLAIR alone. The aim of the MICCAI 2021 MSSEG-2 challenge was to develop an algorithm only based on two longitudinal FLAIRs. Thus, our present work only uses FLAIR as input and a comparison with a multicontrast input is left for future work. Another important aspect that remains to be studied is generalizability to other acquisition settings. In the MICCAI MSSEG-2 challenge, there was quite a variety of different MRI machines. Furthermore, it is interesting to note that the General Electric machines present in the MICCAI MSSEG-2 dataset were not present in the training dataset. However, further experiments, which could not be performed within the challenge setting, would be required to demonstrate generalizability across acquisition settings. Future work will be to go further into dealing with class imbalance during training with a fixed oversampling strategy, as it gave interesting results on the validation set and in other pipelines of the challenge. The difficulty with a fixed oversampling strategy is the arbitrary choice of the oversampling factor. Perhaps inserting neurological priors to guide the oversampling factors and adapting them to the anatomical region could be a promising idea, allowing to take into account the complexity of prediction in some brain areas and the variability of the lesion load over brain regions in MS to tune locally the probability of patches from those regions to be oversampled.

## Conclusion

In this paper, we described our contribution to the MICCAI MSSEG-2 challenge (Commowick et al., 2021). The main new methodological component was the use of online hard example mining (OHEM) for handling class imbalance. Overall, on the challenge testing set, our pipeline ranked at the mid-class, with an average Dice of 0.400 and an average F_1_ score of 0.446. For this specific application, on the validation set, OHEM did not provide any improvement over a standard fixed oversampling strategy. Nevertheless, such a strategy may deserve further investigation for medical imaging problems with class imbalance.

## Data availability statement

The datasets presented in this study can be found in online repositories. The names of the repository/repositories and accession number(s) can be found below: https://portal.fli-iam.irisa.fr/msseg-2/data/.

## Ethics statement

The studies involving human participants were reviewed and approved by OFSEP: https://www.ofsep.org/en. The patients/participants provided their written informed consent to participate in this study.

## Author contributions

MS-M and TS contributed to the pipeline implementation and the manuscript redaction. MH and AY-P contributed to algorithm training, submission, and manuscript redaction. BB and BS contributed with clinical advice and revision. NA, BS, and OC contributed with implementation advice, work supervision, and manuscript revision. All authors contributed to the article and approved the submitted version.

## Funding

The research leading to these results has received funding from the French government under management of Agence Nationale de la Recherche as part of the Investissements d'avenir program, reference ANR-19-P3IA-0001 (PRAIRIE 3IA Institute), reference ANR-10-IAIHU-06 (Agence Nationale de la Recherche-10-IA Institut Hospitalo-Universitaire-6), and reference number ANR-19-P3IA-0002 (3IA Côte d'Azur) and from ICM under the Big Brain Theory program (project IMAGIN-DEAL in MS-M). This work was supported by the Fondation pour la Recherche Médicale, Grant No. FDM202006011247 to TS and by the Fondation Sorbonne Université to MH.

## Conflict of interest

The authors declare that the research was conducted in the absence of any commercial or financial relationships that could be construed as a potential conflict of interest.

## Publisher's note

All claims expressed in this article are solely those of the authors and do not necessarily represent those of their affiliated organizations, or those of the publisher, the editors and the reviewers. Any product that may be evaluated in this article, or claim that may be made by its manufacturer, is not guaranteed or endorsed by the publisher.

## Appendix

We used the MICCAI MSSEG-2 dataset (Commowick et al., 2021), consisting in 100 MS patients with two longitudinal FLAIR MRI spaced from 1 to 3 years, acquired with 6 Philips scanners (Ingenia 1.5T, 2 Ingenia 3T, 1 Achieva dStream 3T, 1 Achieva 1.5T, 1 Achieva 3T), 6 Siemens scanners (1 Aera 1.5T, 1 Skyra 3T, 1 Verio 3T, 1 Prisma 3T, 2 Avanto 1.5T), and 3 General Electrics (GE) scanners (Optima MR450w 1.5T, SIGNA HDx 3T, SIGNA HDxt 1.5T), with different voxel sizes (from 0.5 to 1.2 mm^3^). Ground truth, consisting in new lesions on second time point, were delineated by 4 neuroradiologists from different centers manually on ITK-SNAP (http://www.itksnap.org/pmwiki/pmwiki.php), and consensus was obtained with the majority voting for each voxel. The whole dataset was divided by MSSEG-2 training committee into 40 patients available to challengers for training and validation, and 60 patients, not available to the challengers, for testing. All MRIs acquired with GE were only in the testing dataset.

## References

[B1] Altay E. E. Fisher E. Jones S. E. Hara-Cleaver C. Lee J. C. Rudick R. A.. (2013). Reliability of classifying multiple sclerosis disease activity using magnetic resonance imaging in a multiple sclerosis clinic. JAMA Neurol. 70, 338. 10.1001/2013.jamaneurol.211 23599930 PMC3792494

[B2] Bian C. Yang X. Ma J. Zheng S. Liu Y. A. Nezafat R.. (2022). “Pyramid network with online hard example mining for accurate left atrium segmentation,” in International Workshop on Statistical Atlases and Computational Models of the Heart, (Cham: Springer), 237–245. 10.1007/978-3-030-12029-0_26

[B3] Bodini B. Veronese M. García-Lorenzo D. Battaglini M. Poirion E. Chardain A.. (2016). Dynamic Imaging of Individual Remyelination Profiles in Multiple Sclerosis. Ann. Neurol. 79, 726–738. 10.1002/ana.24620 26891452 PMC5006855

[B4] Cabezas M. Corral J. F. Oliver A. Díez Y. Tintoré M. Auger C.. (2016). Improved automatic detection of new t2 lesions in multiple sclerosis using deformation fields. Am. J. Neuroradiol. 37, 1816–1823. 10.3174/ajnr.A4829 27282863 PMC7960461

[B5] Çiçek Ö. Abdulkadir A. Lienkamp S. S. Brox T. Ronneberger O. (2022). 3D U-Net: Learning Dense Volumetric Segmentation from Sparse Annotation. Published online June 21, 2016. Available online at: http://arxiv.org/abs/1606.06650 (accessed June 28, 2022).

[B6] Commowick O. Cervenansky F. Cotton F. Dojat M. (2021). “MSSEG-2 challenge proceedings: Multiple sclerosis new lesions segmentation challenge using a data management and processing infrastructure,” in MICCAI 2021-24th International Conference on Medical Image Computing and Computer Assisted Intervention, 1–118.

[B7] Commowick O. Istace A. Kain M. Laurent B. Leray F. Simon M.. (2018). Objective evaluation of multiple sclerosis lesion segmentation using a data management and processing infrastructure. Sci. Rep. 8, 13650. 10.1038/s41598-018-31911-7 30209345 PMC6135867

[B8] Filippi M. Brück W. Chard D. Fazekas F. Geurts J. J. Enzinger C.. (2019). Association between pathological and MRI findings in multiple sclerosis. Lancet Neurol. 18, 198–210. 10.1016/S1474-4422(18)30451-4 30663609

[B9] Gessert N. Krüger J. Opfer R. Ostwaldt A. C. Manogaran P. Kitzler H. H.. (2020). Multiple sclerosis lesion activity segmentation with attention-guided two-path CNNs. Comput. Med. Imaging Graph. 84, 101772. 10.1016/j.compmedimag.2020.101772 32795845

[B10] He K. Fan H. Wu Y. Xie S. Girshick R. (2020). “Momentum contrast for unsupervised visual representation learning,” in 2020 IEEE/CVF Conference on Computer Vision and Pattern Recognition (CVPR) (IEEE), 9726–9735. 10.1109/CVPR42600.2020.00975

[B11] Hegen H. Bsteh G. Berger T. (2018). ‘No evidence of disease activity' - is it an appropriate surrogate in multiple sclerosis? Eur. J. Neurol. 25, 1107–e101. 10.1111/ene.13669 29687559 PMC6099351

[B12] Howard J. Trevick S. Younger D. S. (2016). Epidemiology of multiple sclerosis. Neurol. Clin. 34, 919–939. 10.1016/j.ncl.2016.06.016 27720001

[B13] Jenkinson M. Smith S. (2001). A global optimisation method for robust affine registration of brain images. Med. Image Anal. 5, 143–156. 10.1016/S1361-8415(01)00036-6 11516708

[B14] Kingma D. P. Ba J. (2022). Adam: A Method for Stochastic Optimization. Published online January 29, 2017. Available online at: http://arxiv.org/abs/1412.6980 (accessed June 28, 2022).

[B15] McKinley R. Wepfer R. Grunder L. Aschwanden F. Fischer T.. (2020). Automatic detection of lesion load change in Multiple Sclerosis using convolutional neural networks with segmentation confidence. NeuroImage Clin. 25, 102104. 10.1016/j.nicl.2019.102104 31927500 PMC6953959

[B16] Paszke A. Gross S. Chintala S. Chanan G. Yang E. DeVito Z.. (2017). “Automatic differentiation in PyTorch,” in NIPS 2017 Workshop Autodiff Submission.

[B17] Pérez-García F. Sparks R. Ourselin S. (2021). TorchIO: A Python library for efficient loading, preprocessing, augmentation and patch-based sampling of medical images in deep learning. Comput. Methods Progr. Biomed. 208, 106236. 10.1016/j.cmpb.2021.106236 34311413 PMC8542803

[B18] Salem M. Valverde S. Cabezas M. Pareto D. Oliver A.. (2020). A fully convolutional neural network for new T2-w lesion detection in multiple sclerosis. NeuroImage Clin. 25, 102149. 10.1016/j.nicl.2019.102149 31918065 PMC7036701

[B19] Shrivastava A. Gupta A. Girshick R. (2016). “Training region-based object detectors with online hard example mining,” in 2016 IEEE Conference on Computer Vision and Pattern Recognition (CVPR) (IEEE), 761–769. 10.1109/CVPR.2016.89

[B20] Valverde S. Salem M. Cabezas M. Pareto D. Vilanova J. C. Ramió-Torrentà L.. (2019). One-shot domain adaptation in multiple sclerosis lesion segmentation using convolutional neural networks. NeuroImage Clin. 21, 101638. 10.1016/j.nicl.2018.101638 30555005 PMC6413299

[B21] Wang X. Ma H. Chen X. (2016). “Salient object detection via fast R-CNN and low-level cues,” in 2016 IEEE International Conference on Image Processing (ICIP) (IEEE), 1042–1046. 10.1109/ICIP.2016.7532516

[B22] Wolny A. Cerrone L. Vijayan A. Tofanelli R. Barro A. V. Louveaux M.. (2020). Accurate and versatile 3D segmentation of plant tissues at cellular resolution. eLife. 9, e57613. 10.7554/eLife.57613 32723478 PMC7447435

[B23] Zeng C. Gu L. Liu Z. Zhao S. (2020). Review of Deep Learning Approaches for the Segmentation of Multiple Sclerosis Lesions on Brain MRI. Front Neuroinformatics. 14, 610967. 10.3389/fninf.2020.610967 33328949 PMC7714963

